# Body mass index, waist-to-hip ratio and late outcomes: a report from the Shanghai Breast Cancer Survival Study

**DOI:** 10.1038/s41598-017-07320-7

**Published:** 2017-08-01

**Authors:** Minlu Zhang, Hui Cai, Pingping Bao, Wanghong Xu, Guoyou Qin, Xiao Ou Shu, Ying Zheng

**Affiliations:** 1 Department of Biostatistics, School of Public Health and Key Laboratory of Public Health Safety, Fudan University, Shanghai, People’s Republic of China; 2 Department of Cancer Control and Prevention, Shanghai Municipal Center for Disease Control and Prevention, Shanghai, People’s Republic of China; 3 Division of Epidemiology, Department of Medicine, Vanderbilt University School of Medicine, Nashville, USA; 4 Department of Chronic Non-Communicable Disease Surveillance, Shanghai Municipal Center for Disease Control and Prevention, Shanghai, People’s Republic of China; 5 Department of Epidemiology, School of Public Health and Key Laboratory of Public Health Safety, Fudan University, Shanghai, People’s Republic of China; 6 Collaborative Innovation Center of Social Risks Governance in Health, Fudan University, Shanghai, People’s Republic of China; 7 Present Address: Department of Cancer Prevention, Shanghai Cancer Center, Fudan University, Shanghai, People’s Republic of China

## Abstract

Obesity has been well studied in relation to breast cancer survival. However, the associations of post-diagnosis obesity and late outcomes (≥5 years after diagnosis) have been much less studied. A total of 4062 5-year disease-free patients were recruited from the Shanghai Breast Cancer Survival Study, a longitudinal study of patients diagnosed during 2002-2006. Cox proportional hazard model with restricted cubic spline were used to evaluate the potential non-linear associations of post-diagnosis body mass index (BMI) and waist-to-hip ratio (WHR) with late all-cause mortality and late recurrence. While no significant association was observed for post-diagnosis BMI or WHR with late recurrence; a U-shaped association was observed for the two measures with late all-cause death. Women with BMI of 25.0 kg/m^2^ or WHR of 0.83 were at the lowest risk of late all-cause mortality, whereas those with BMI beyond the range of 22.1–28.7 kg/m^2^ or WHR beyond the range of 0.81–0.86 had a higher risk. ER, stage or menopausal status did not modify the effect of post-diagnosis BMI or WHR on the outcomes. In conclusion, post-diagnosis BMI and WHR, as indicators of overall and central obesity respectively, were associated with late all-cause mortality in U-shaped pattern among long-term breast cancer survivors.

## Introduction

Body mass index (BMI) has been consistently associated with both all-cause mortality and recurrence in a U-shaped or J-shaped pattern^1–6^. By contrast, waist-to-hip ratio (WHR), an index of central obesity, was much less studied, and the results were mixed. Most studies^1, 2, 7–9^, not all^3, 10^ suggested that WHR was positively associated with breast cancer survival. With the increasing 5-year survival rate in breast cancer patients around the world, including China^11^, more concern has been aroused on the role of obesity in late outcomes of breast cancer. Breast cancer patients may have a considerable residual risk of recurrence in later years, especially in estrogen receptor (ER)-positive patients who were treated with adjuvant endocrine therapy^12, 13^.

Several studies had divided events into those occurred within and after 5 or 10 years after diagnosis to identify prognostic factors of late outcomes, such as obesity, tumor size, nodal status, tumor grade and recurrence score^14–19^. Among them, only two studies investigated the associations between obesity and late outcomes^14, 19^. In the two studies, obesity (BMI ≥ 30 kg/m^2^) or severe obesity (BMI ≥ 35 kg/m^2^) post-diagnosis or at diagnosis were found in relation to higher hazard of all-cause death, breast cancer death and recurrence 5–10 years after diagnosis. However, no studies have ever evaluated the association between WHR and late outcomes.

In our previous report based on the Shanghai Breast Cancer Survival Study (SBCSS), we found that obesity (BMI > 30 kg/m^2^) measured at 6 months after diagnosis was inversely related to breast cancer prognosis after a median follow-up time of 46 months (i.e. early outcomes)^3^, but did not observe a significant association for WHR. In this study, we further followed up the cancer cases to explore the relationship of both indicators with late all-cause mortality and late recurrence, i.e. events occurring 5 years after diagnosis. In addition to using a traditional analysis method, which categorizes BMI and WHR according to World Health Organization standard^20^ or quartile distributions, we applied Cox proportional hazard model with restricted cubic spline (RCS) to reveal the potential non-linearity associations of BMI and WHR with late outcomes.

## Results

### Demographic, clinical and lifestyle factors in 5-year disease-free patients

Among 4062 5-year disease-free patients, 326 deaths and 264 recurrences occurred ≥ 5 years after diagnosis. The median follow-up time for late all-cause mortality and late recurrence were 10.54 years (5.02–12.78) and 8.40 (5.01–11.03), respectively.

Table 1 shows demographic characteristics, clinical predictors and lifestyle factors of 5-year disease-free patients assessed at 6 months after diagnosis, as well as BMI, WHR and menopausal status measured at 60 months’ post-diagnosis. In the patients, 73.4% were at early stage (I-IIA) of breast cancer, more than 65.5% were ER-positive, 5.0% were general obese and 39.3% were central obese at baseline. BMI and WHR at 6 months’ post-diagnosis were significantly correlated, with a Pearson correlation coefficient of 0.47 (*P* < 0.001). Pearson correlation coefficient demonstrated that WHR was more correlated with waist circumference (*r* = 0.75) than hip circumference (*r* = 0.25).

**Table 1:** Demographic characteristics, clinical and lifestyle factors assessed at the 6 months’ post-diagnosis in 5-year disease-free patients, the Shanghai Breast Cancer Survival Study.

Characteristics	*N* = 4062	
	Frequency	%
Age at diagnosis ($\overline{{\rm{x}}}$ ± SD, year)	53.2 ± 9.9	
Soy protein intake ($\overline{{\rm{x}}}$ ± SD, g/d)	12.0 ± 8.9	
BMI ($\overline{{\rm{x}}}$ ± SD, kg/m^2^)	24.0 ± 3.3	
<21.88	1070	26.3
21.88–23.9	1085	26.7
24–26.3	1009	24.8
≥26.3	898	22.1
WHR (quartile) ($\overline{{\rm{x}}}$ ± SD)	0.8 ± 0.1	
<0.80	989	24.4
0.80–0.82	884	21.8
0.83–0.86	1129	27.8
≥0.87	1060	26.1
Exercise participation^a^	2668	65.7
Education level		
<High school	1823	44.9
High school	1568	38.6
>High school	671	16.5
Post-menopausal^a^	2033	50.1
Charlson index of comorbidity ≥1^a^	780	19.2
Mastectomy^a^	3840	94.5
Chemotherapy^a^	3747	92.3
Radiotherapy^a^	1236	30.4
ER status		
Positive	2662	65.5
Negative	1356	33.4
Unknown	44	1.1
PR status		
Positive	2408	59.3
Negative	1597	39.3
Unknown	57	1.4
TNM stage		
I	1546	38.1
IIA	1433	35.3
IIB	631	15.5
III-IV	262	6.5
Unknown	190	4.7

^a^Compared with women who had no corresponding characteristics.

### Associations of BMI and WHR with late outcomes

Table 2 presents the associations of BMI and WHR as categorical variables with late outcomes of breast cancer. After adjusting for demographic, clinical and lifestyle factors, no significant associations were observed for BMI and WHR with later recurrence; but U-shaped associations were observed for BMI and WHR with late all-cause mortality.

**Table 2:** Hazard ratios and 95% confidence intervals for post-diagnosis BMI and WHR in association with late all-cause mortality and recurrence of breast cancer, the Shanghai Breast Cancer Survival Study.

Predictors	No. of subjects	Late all-cause mortality			Late recurrence		
		No. of events	HR	95% CI	No. of events	HR	95% CI
	4062	326			264		
BMI^a^							
<21.88	1070	64	1.16	0.84–1.60	52	0.72	0.50–1.05
21.88–23.9	1085	84	1.00		80	1.00	
24–26.3	1009	76	0.93	0.67–1.29	62	0.95	0.68–1.33
≥26.3	898	102	1.10	0.81–1.49	70	1.02	0.73–1.42
BMI^b^							
<21.88	1070	64	1.13	0.82–1.57	52	0.71	0.49–1.03
21.88–23.9	1085	84	1.00		80	1.00	
24–26.3	1009	76	0.92	0.66–1.27	62	0.96	0.68–1.35
≥26.3	898	102	1.05	0.77–1.44	70	1.01	0.72–1.42
WHR^a^							
<0.80	989	64	1.46	1.03–2.08	58	1.04	0.72–1.50
0.80–0.82	884	60	1.00		55	1.00	
0.83–0.86	1129	85	1.06	0.75–1.49	70	0.71	0.49–1.02
≥0.87	1060	117	1.43	1.03–1.99	81	1.18	0.84–1.66
WHR^c^							
<0.80	989	64	1.42	0.99–2.03	58	1.07	0.74–1.56
0.80–0.82	884	60	1.00		55	1.00	
0.83–0.86	1129	85	1.07	0.76–1.51	70	0.70	0.48–1.01
≥0.87	1060	117	1.45	1.04–2.03	81	1.13	0.80–1.60

^a^Adjusted for age at diagnosis, soy protein intake, regular exercise, ER status, TNM stage, mastectomy, chemotherapy, radiotherapy, comorbidity, education and menopausal status. ^b^Additionally adjusted for WHR (<0.80/0.80–0.82/0.83–0.86/≥0.87). ^c^additionally adjusted for BMI (<21.88/21.88–23.9/24–26.3/≥26.3).

**Figure 1:**
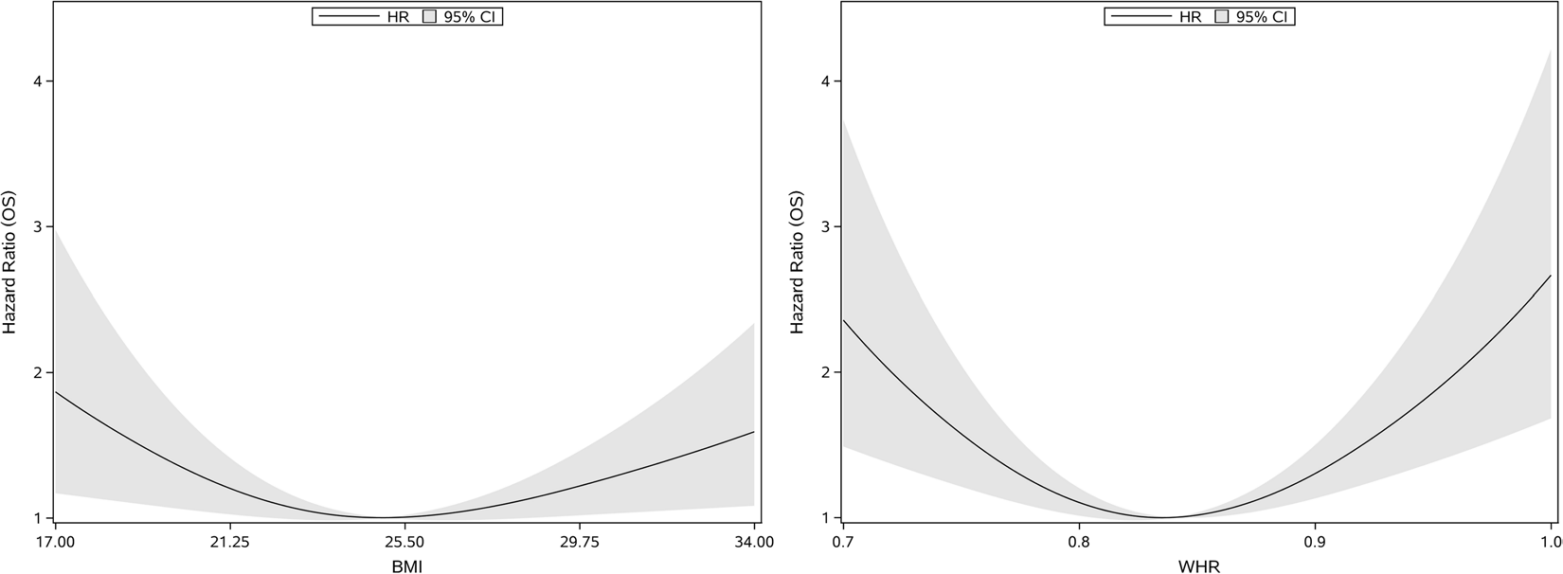
Association of post-diagnosis (**A**) BMI and (**B**) WHR with late all-cause mortality. The graphs were truncated at the 1st and 99th percentiles with curve representing HRs and band representing 95% CI.

Further analysis in Cox models with RCS found that both BMI and WHR were significantly related to late all-cause mortality (*P* = 0.01 and <0.001 respectively) (Table 3). The associations were in nonlinear U-shaped pattern (*P* = 0.003 and <0.001 respectively), and patients with BMI beyond (below or above) the range of 22.1–28.7 kg/m^2^ having a higher risk of death (Fig. 1a and b). Compared with patients with BMI of 25.0 kg/m^2^ (reference BMI with lowest hazard), those with BMI at 1^st^ percentile (near 17.0 kg/m^2^) had 86% increased risk (95% CI: 1.17–2.98), and those with BMI at 99^th^ percentile (near 34.0 kg/m^2^) had 59% increased risk (95% CI: 1.08–2.34). Further adjustment for WHR did not substantially change the pattern. A similar but more pronounced association pattern was observed for WHR with late all-cause mortality. However, as shown in Table 3, no significant association was observed for BMI and WHR with late recurrence (*P* = 0.240 and 0.054, respectively).

**Table 3:** Hazard ratios and 95% confidence intervals for post-diagnosis BMI and WHR in association with late all-cause mortality and recurrence of breast cancer, the Shanghai Breast Cancer Survival Study.

Factors	Late all-cause mortality					Late recurrence				
	Value	HR	95% CI	*P* for overall association	*P* for non-linearity	Value	HR	95% CI	*P* for overall association	*P* for non-linearity
BMI^a^										
1^st^ percentile	17.0	1.86	1.17–2.98	0.010	0.003	17.0	0.80	0.44–1.44	0.240	0.966
25^th^ percentile	22.0	1.13	1.01–1.28			22.0	0.91	0.79–1.06		
reference^d^	25.0	1.00				25.0	1.00			
75^th^ percentile	26.0	1.01	0.98–1.04			26.0	1.03	0.99–1.07		
99^th^ percentile	34.0	1.59	1.08–2.34			34.0	1.32	0.81–2.14		
BMI^b^										
1^st^ percentile	17.0	2.02	1.23–3.29	0.008	0.002	17.0	0.87	0.47–1.62	0.506	0.875
25^th^ percentile	22.0	1.16	1.02–1.33			22.0	0.94	0.80–1.10		
reference^d^	25.3	1.00				25.3	1.00			
75^th^ percentile	26.0	1.01	0.98–1.03			26.0	1.02	0.99–1.05		
99^th^ percentile	34.0	1.52	1.03–2.25			34.0	1.25	0.77–2.03		
WHR^a^										
1^st^ percentile	0.7	2.36	1.49–3.73	<0.001	<0.001	0.7	1.23	0.71–2.12	0.054	0.082
25^th^ percentile	0.80	1.11	1.01–1.32			0.80	1.01	0.94–1.08		
reference^d^	0.84	1.00				0.82	1.00			
75^th^ percentile	0.87	1.10	1.03–1.17			0.87	1.12	1.00–1.25		
99^th^ percentile	1.0	2.66	1.68–4.22			1.0	2.03	1.14–3.61		
WHR^c^										
1^st^ percentile	0.7	2.32	1.45–3.73	<0.001	<0.001	0.7	1.32	0.76–2.28	0.123	0.079
25^th^ percentile	0.80	1.10	1.00–1.21			0.80	1.02	0.95–1.09		
reference^d^	0.84	1.00				0.82	1.00			
75^th^ percentile	0.87	1.10	1.03–1.17			0.87	1.08	0.96–1.22		
99^th^ percentile	1.0	2.71	1.68–4.36			1.0	1.84	1.01–3.34		

^a^Adjusted for age at diagnosis, soy protein intake, ER status, TNM stage, mastectomy, chemotherapy, radiotherapy, regular exercise, comorbidity, education and menopausal status.

^b^Additionally adjusted for WHR as a continuous variable.

^c^Additionally adjusted for BMI as a continuous variable.

^d^Using the BMI or WHR value with the lowest hazard as reference.

### Associations of BMI and WHR with late outcomes by ER status, TNM stage and menopausal status

In further stratified analysis, the relationship between BMI and late all-cause mortality did not vary greatly by ER status, TNM stage and menopausal status (*P* for interaction = 0.327, 0.610 and 0.569 respectively), although the U-shaped associations appeared more pronounced in ER-negative and postmenopausal patients, and the association of lower BMI with higher hazard was more prominent in patients with stage I breast cancer (Table 4). In addition, ER status itself was a significant prognostic factor, with ER-positive status related with 36% (95% CI: 1.06–1.74) higher risk of late all-cause mortality.

**Table 4:** Hazard ratios and 95% confidence intervals for post-diagnosis BMI in association with late all-cause mortality by ER status and menopausal status, the Shanghai Breast Cancer Survival Study.

BMI	No. of cases	All-cause deaths	HR	95% CI	*P* for overall association	*P* for non-linearity	*P* for interaction
ER status							
ER-negative	1356	88			0.014	0.003	0.327
25.3 (ref)			1.00				
17.0 (1^st^ percentile)			2.93	1.33–6.48			
22.0 (25^th^ percentile)			1.27	1.02–1.58			
26.0 (75^th^ percentile)			1.01	0.97–1.04			
34.0 (99^th^ percentile)			1.76	1.00–3.10			
ER-positive	2662	233			0.278	0.113	
24.7 (ref)			1.00				
17.0 (1^st^ percentile)			1.48	0.82–2.66			
22.0 (25^th^ percentile)			1.08	0.93–1.24			
26.0 (75^th^ percentile)			1.01	0.97–1.06			
34.0 (99^th^ percentile)			1.42	0.85–2.38			
TNM stage							
Stage I	1546	74			0.010	0.005	0.610
25.5 (ref)			1.00				
17.0 (1^st^ percentile)			3.28	1.48–7.24			
22.0 (25^th^ percentile)			1.31	1.05–1.65			
26.0 (75^th^ percentile)			1.00	0.97–1.04			
34.0 (99^th^ percentile)			1.76	0.85–3.67			
Stage II	2064	182			0.226	0.143	
24.1 (ref)			1.00				
17.0 (1^st^ percentile)			1.36	0.72–2.57			
22.0 (25^th^ percentile)			1.05	0.91–1.20			
26.0 (75^th^ percentile)			1.03	0.96–1.10			
34.0 (99^th^ percentile)			1.54	0.93–2.56			
Stage III- IV	262	52			0.387	0.170	
24.8 (ref)			1.00				
17.0 (1^st^ percentile)			2.15	0.59–7.90			
22.0 (25^th^ percentile)			1.16	0.84–1.59			
26.0 (75^th^ percentile)			1.02	0.93–1.13			
34.0 (99^th^ percentile)			1.93	0.62–5.98			
Menopausal status							
Postmenopausal	3361	293			0.030	0.009	0.569
24.8 (ref)			1.00				
17.0 (1^st^ percentile)			1.73	1.04–2.88			
22.0 (25^th^ percentile)			1.11	0.98–1.26			
26.0 (75^th^ percentile)			1.02	0.98–1.05			
34.0 (99^th^ percentile)			1.58	1.06–2.35			

As shown in Table 5, a borderline significant modifying effect was observed for ER status in the association between WHR and late all-cause mortality (*P* for interaction = 0.087). Among ER-negative patients, comparing with those having the lowest risk (WHR of 0.85), women with WHR of 0.70 had 4-fold risk of late mortality, and women with WHR of 1.0 had a doubled risk. In ER-positive patients, on the other hand, comparing with those having the lowest risk (WHR of 0.83), women with WHR of 0.70 and 1.0 had 44% increased risk and nearly 3-fold risk of late mortality, respectively.

**Table 5:** Hazard ratios and 95% confidence intervals for post-diagnosis WHR in association with late all-cause mortality by ER status and menopausal status, the Shanghai Breast Cancer Survival Study.

WHR	No. of cases	All-cause deaths	HR	95% CI	*P* for overall association	*P* for non-linearity	*P* for interaction
ER status							
ER-negative	1356	88			<0.001	<0.001	0.087
0.85 (ref)			1.00				
0.7 (1^st^ percentile)			4.38	2.21–8.69			
0.8 (25^th^ percentile)			1.29	1.10–1.53			
0.87 (75^th^ percentile)			1.03	0.94–1.14			
1.0 (99^th^ percentile)			2.09	0.82–5.37			
ER-positive	2662	233			0.001	0.002	
0.83 (ref)			1.00				
0.7 (1^st^ percentile)			1.44	0.93–2.22			
0.8 (25^th^ percentile)			1.04	0.95–1.15			
0.87 (75^th^ percentile)			1.14	1.04–1.25			
1.0 (99^th^ percentile)			2.79	1.62–4.82			
TNM stage							
Stage I	1546	74			0.046	0.031	0.996
0.84 (ref)			1.00				
0.7 (1^st^ percentile)			2.66	1.21–5.84			
0.8 (25^th^ percentile)			1.16	0.99–1.35			
0.87 (75^th^ percentile)			1.05	0.90–1.22			
1.0 (99^th^ percentile)			2.05	0.66–6.36			
Stage II	2064	182			<0.001	<0.001	
0.84 (ref)			1.00				
0.7 (1^st^ percentile)			2.46	1.30–4.64			
0.8 (25^th^ percentile)			1.11	0.98–1.26			
0.87 (75^th^ percentile)			1.10	1.02–1.19			
1.0 (99^th^ percentile)			2.85	1.60–5.07			
Stage III- IV	262	52			0.294	0.142	
0.83 (ref)			1.00				
0.7 (1^st^ percentile)			2.05	0.55–7.66			
0.8 (25^th^ percentile)			1.08	0.87–1.34			
0.87 (75^th^ percentile)			1.10	0.91–1.35			
1.0 (99^th^ percentile)			2.58	0.74–9.02			
Menopausal status							
Postmenopausal	3361	293			<0.001	<0.001	0.185
0.84 (ref)			1.00				
0.7 (1^st^ percentile)			2.24	1.35–3.70			
0.8 (25^th^ percentile)			1.10	0.99–1.21			
0.87 (75^th^ percentile)			1.10	1.03–1.17			
1.0 (99^th^ percentile)			2.68	1.68–4.30			

Due to small sample size (436) and number of events (11) in premenopausal women, we could not evaluate the potential modifying effect of menopausal status on the U-shape associations of WHR and BMI with later all-cause mortality. No significant modifying effect was observed for age at diagnosis (<65/ ≥ 65 years old), physical activity (Yes/No) and comorbidity (Yes/No), either.

## Discussion

In this cohort study of 4062 5-year disease-free patients, we found that BMI and WHR were associated with late all-cause mortality in a U-shaped pattern. The lowest hazard was observed in patients with BMI of 25.0 kg/m^2^ or in patients with WHR of 0.84, and a higher risk was found in patients with BMI beyond the range of 22.1–28.7 kg/m^2^ or in those with WHR beyond the range of 0.81–0.86. Neither BMI nor WHR was related to late recurrence. To our knowledge, this study is the first one to evaluate the effect of WHR on late outcomes in long-term breast cancer survivors.

Our finding of the U-shaped association of BMI with late all-cause mortality was consistent with some of the previous studies^14, 19^, in which one study in Denmark found that positive association of obesity (BMI ≥ 30 kg/m^2^) at diagnosis with the risk of distant metastasis or breast cancer death were stronger beyond than within 5 years after diagnosis^19^. In a pooled analysis on ER-positive breast cancer^14^, U-shaped and J-shaped associations were detected for 4.6 years’ post-diagnosis BMI with late all-cause mortality and late recurrence, respectively. Our findings, as well as the positive association of obesity with early outcome of breast cancer previously reported in this population^3^, provide strong support for the predictive role of obesity in long-term survival of breast cancer patients.

Obesity has been consistently found a negative prognostic factor in breast cancer patients, but the effect of underweight remains unclear. In this study, we found that women with BMI <22.1 kg/m^2^, including those underweighted and patients with normal-low BMI, had elevated risk of death. As all patients in our study were long-term survivors who went through long course of disease, the inhibited immune system and cytokine reactions caused by chronic malnutrition^21^, and weight loss resulted from illness might explain the result. Unintentional weight loss caused by cachexia could be the reason for the association between lower BMI and increased risk of death. However, cachexia was not documented in our study and we were not able to evaluate its role in the mechanism. Moreover, lower BMI could be due to preexisting comorbidities that had already placed these women at greater risk of poor outcomes. In our study, we adjusted for comorbidity as Charlson comorbidity index ≥ 1 vs. 0, where index ≥ 1 meant the patient had at least one comorbid condition among the scoring range of Charlson comorbidity index, and the association estimates did not differ by the two strata. Further analysis using original continuous scores of Charlson comorbidity did not change the lower BMI/WHR-mortality associations. However, residual confounding by other unmeasured comorbidities outside the scoring range of Charlson comorbidity index could be possible. It’s noteworthy that the negative prognostic effect of underweight was more likely to be observed in Asian populations^4–6, 22^. In 24,698 Korean breast cancer patients, underweighted (<18.5 kg/m^2^) patients were observed to have a significantly poorer overall survival and shorter time to distant and local recurrence compared with patients with normal weight (18.5–24.9 kg/m^2^)^5^. The study conducted in 20,090 Japanese cases also observed a significant underweight-mortality association in all and postmenopausal breast cancer patients^22^.

Comparing with BMI, WHR, as an approximation of visceral adipose tissue, is a better measurement for central obesity and elderly people^23^. However, the prognostic effect of WHR in breast cancer was much less studied. While several long-term follow-up studies (more than 10 years) reported an increased risk of all-cause death in the highest vs. the lowest quartile groups of WHR^1, 2, 7–9, 24^, the other two studies with follow-up time around 5 years did not find a significant association^3, 10^. In this study, we observed, for the first time, a U-shaped association between WHR and late all-cause mortality, in which a higher or a lower WHR was related with a higher risk of death. In the former report of our cohort, HR in each quartile of WHR also presented a U-shaped pattern, although the association was not significant^3^. This indicated that the lack of significance might be attributable to using traditional analysis method by categorizing predictor, which reduced statistical power.

The mechanisms underlying the U-shaped pattern are unclear. 82.7% of the women in our study were post-menopausal at about 5 years after their diagnosis. They tend to lose lean body mass and have a shift of body fat from peripheral to central sites with an accompanying increase in WHR at the same level of BMI^23^. Higher WHR representing higher VAT is related to insulin resistance, hyperinsulinemia, adipose-derived hormones and chronic inflammation, which are thought to play an important role in carcinogenesis^25, 26^. Insulin further stimulates the production of estrogen and the expression of ER-α in breast cancer cells^27^. As to the patients with low WHR, it is speculated that inhibited immune system caused by undernutrition, which justify the association of lower BMI with higher mortality might also be applicable to the link between low WHR and higher mortality. Due to limited studies on WHR, these associations need to be verified in future studies, especially in Asians.

Results of studies evaluating the effect of BMI according to hormone receptor status were inconsistent. It has been indicated that the effect of general obesity on breast cancer prognosis may be stronger in women with ER-positive tumors than women with ER-negative tumors^28, 29^. But, a meta-analysis of 21 studies found no evidence that the relation of obesity to breast cancer outcomes varies by hormone receptor status^30^. Moreover, results from the randomized SUCCESS A trial demonstrated that the adverse effect of severe obesity was only found in triple negative breast cancer subgroup rather than luminal or HER2-positive subtypes^31^. In our study, the association appeared to be more apparent in ER-negative patients in our study, although the interaction was not significant. It might be speculated that the association in ER-positive patients could have been masked by the benefit of endocrine therapies such as tamoxifen or aromatase inhibitor.

Positive relation of WHR to breast cancer mortality was restricted to ER-positive postmenopausal women in a study of Vancouver, Canada^8^, whereas no modifying effect was found in other two studies^1, 2^. Our study suggested a significant association of WHR with late all-cause mortality among both women with ER-negative and ER-positive breast cancer. The lower WHR-mortality relation was more obvious in ER-negative patients; while the higher WHR-mortality relation was more apparent in ER-positive patients, where the estrogen mediated mechanism could justify this association^8^.

Recent studies observed varying associations between BMI and survival of colorectal cancer^32^, ovarian cancer^33^ and many other types of cancer^34^ across TNM stages. As to breast cancer, an Italian study observed an unfavorable effect of high BMI only in women with stage I-II breast cancer^1^. However, no formal interaction test was performed and the sample size was small in advanced-stage patients. Another study found that the association was consistent across strata of cancer stages^35^. In our study, although the interaction test was not significant, we observed a significant association between BMI and late all-cause mortality in stage I patients, but not among those with stage III-IV cancer. However, the null association in the stage III-IV patients could be due to small sample size and rare events in this stratum. Further study involving larger number of advanced breast cancers is warranted. In addition, BMI-mortality association was more pronounced in patients with lower BMI than in patients with higher BMI in stage I patients. This could be due to the fact that patients with higher BMI tend to be diagnosed with later stage^35^. Interestingly, in line with previous studies conducted in US populations^36, 37^, we find that the effect of ER status in breast cancer survival was also time-dependent in Chinese patients. ER-positive status was a protective factor for early outcomes^38^, but a risk factor for late outcomes (≥5 years) which may provide reference for the treatment of breast cancer.

The main strengths of this study included the large sample size, more than 10 years’ follow-up time for all-cause death, detailed information on post-diagnostic lifestyle, clinical factors and accurate anthropometric measurements taken at 6 and 60 months after diagnosis, and use of RCS in data analysis. The RCS, by using all information, increasing statistical power, and testing non-linear relationship, has enabled us to evaluate the risk for each value of BMI and WHR and to identify the lowest risk point of BMI and WHR. However, several limitations should be mentioned. First, the average follow-up time for recurrence was only 8.4 years, relatively shorter than that for all-cause mortality, which may had led to small power to evaluate the association of obesity with risk of recurrence. Moreover, we just treated BMI and WHR as time-dependent variables, but not for other variables including ER status, which may also have changed during the 10-year follow-up time. This may introduce bias to our results.

In summary, our findings of the U-shaped associations between BMI, WHR and late all-cause mortality provide strong evidence on the long-term effect of obesity on breast cancer survival, and indicate the benefits of keeping moderate body size for breast cancer patients. Further studies are warranted to evaluate the potential modifying effect of ER and menopausal status in the associations.

## Methods

### Study population

Patients included in this study were from the SBCSS, a longitudinal, population-based study of women aged 20 to 75 years old who were diagnosed with primary breast cancer between March 2002 and April 2006. Details of the study design of the SBCSS have been described previously^39^. In brief, all patients were permanent residents of Shanghai, China, and from the Shanghai Cancer Registry. Among 6299 patients contacted, 5042 women provided written, informed consent and were recruited into the study approximately 6 months after diagnosis. Reasons for non-participation included: refusal (n = 757, 12.0%), absence during study enrollment (n = 258, 4.1%), unable to contact (n = 83, 1.3%), and other miscellaneous reasons such as health or communication problems (n = 159, 2.5%). 156 patients diagnosed with breast cancer *in situ* were further excluded. Of 4886 subjects, 4062 were 5-year disease-free patients without death/recurrence/loss to follow-up prior to 5 years after diagnosis and were included in the analysis.

### Ethics

This study was approved by the institutional review board and ethic committee of Vanderbilt University and Shanghai Municipal Center for Disease Control and Prevention. The study was carried out in accordance with the relevant guidelines and regulations. All participants in this research provided their written consents.

### Data collection

In-person interviews were conducted approximately 6, 18, 36, and 60 months after diagnosis using questionnaires, with follow-up rates of 91%, 84%, and 77%, respectively, for the 18-, 36-, and 60-month post-diagnosis interview. Information on demographics, cancer diagnosis and treatment, menstrual and reproductive factors, selected life style factors (soy protein intake, cigarette and alcohol use, physical activity, etc.), comorbidity, use of complementary and alternative medicine and quality of life were collected at baseline survey. Medical records were reviewed to collect clinical information on date of diagnosis, TNM stage, ER status, progesterone receptor status, surgery, radiotherapy, chemotherapy, immunotherapy and hormonal therapy. A Charlson comorbidity index covering 19 categories of comorbid conditions was created for each woman based on a validated comorbidity scoring system, with index 0 and ≥1 representing not having and having at least one comorbidity within the scoring categories, respectively^40^. Menopausal status was defined as cessation of menstruation for at least 12 months, excluding situation caused by pregnancy or breast-feeding and hormone-induced menopause.

Anthropometric measurements (height, weight, waist and hip circumference) were taken twice at 6 and 60 months after diagnosis by trained interviewers according to a standard protocol. Self-reported weight at 18 and 36 months after diagnosis were not used in this analysis. Detailed measurements method was described in our previous report^3^. Briefly, weight was measured to the nearest 0.1 kg using a digital weight scale that was calibrated every 6 months. Standing height and circumferences were measured to the nearest 0.1 cm. Waist circumference was measured at 2.5 cm above the umbilicus and hip circumference at the level of maximum width of the buttocks with the subject in a standing position. BMI (weight in kilograms divided by the square of height in meters) and WHR (waist circumference divided by hip circumference) were then calculated based on the measurements. All 4062 patients in this study took the first measurements (6 months’ post-diagnosis), and 3292 and 3304 patients were measured with BMI and WHR, respectively, at 60 months’ post-diagnosis.

Survival status of each participant was collected by data linkage with the Shanghai Vital Statistics database. The most recent linkage was conducted on December 31, 2014 for all-cause mortality and December 31, 2012 for recurrence.

### Statistical analysis

Outcomes for this study were late (≥5 years) all-cause death and late disease-free survival with an event defined as recurrence, metastasis, or breast cancer specific death, whichever occurred first, and referred to as late recurrence for convenience. All the analyses were based on 5-year disease-free survivors, who survived more than 5 years after their cancer diagnosis and without recurrence or metastasis^41^. Survival time was calculated as the period from the date of diagnosis to the date of death (or recurrence for the recurrence analysis) or date of last contact (i.e., date of last follow-up survey or last registry linkage, whichever was most recent).

Cox proportional hazard model was used to estimate the associations of BMI, WHR with late outcomes. Log-log survival plot was applied to evaluate proportional hazard assumption for these two variables. At first, BMI or WHR were put into the model as categorical variables. They were categorized according to quartile distribution instead of World Health Organization classification^20^ in that sample size of underweight group (BMI <18.5 kg/m^2^) was too small to give stable estimation. And then, BMI or WHR were treated as continuous variables in the model, and restricted cubic spline function was utilized to examine non-linearity relationship between predictors and outcomes and visually demonstrate the relationship; knots were placed at the 5^th^, 50^th^, 95^th^ percentiles^42, 43^, values of BMI and WHR with the lowest hazard were taken as reference to estimate hazard ratios (HR) and 95% confidence intervals (CI) for any other values. Two statistical tests were conducted during this procedure, one test was for the null hypothesis that the regression coefficients of both linear and nonlinear terms of the factor were equal to zero, and the result was presented as “*P* for overall association”; another test was for the regression coefficient of nonlinear term, i.e. spline variable, and “*P* for non-linearity” <0.05 indicated a non-linear association. BMI or WHR were included as time-dependent variables by counting process method to capture changes during the follow-up process. Both 6 and 60 months’ post-diagnosis measurements were used if available; otherwise only 6 months’ post-diagnosis measurements were used.

Confounders adjusted in the Cox model included age at diagnosis (continuous), soy protein intake (continuous), ER status (positive/negative/unknown), TNM stage (I/IIA/IIB/III-IV/unknown), mastectomy (yes/no), chemotherapy (yes/no), radiotherapy (yes/no), comorbidity (0/ ≥ 1), physical activity (yes/no), education (<high school/high school/ > high school) and menopausal status (premenopausal/postmenopausal/unknown). Binary variables entered the model directly and polytomous variables were treated as dummy variables. Soy protein intake and physical activity was assessed at 6 months’ post-diagnosis, and menopausal status was at 60 months’ post-diagnosis as many patients changed their status during the follow-up. Smoking and alcohol drinking rates were very low in our study population, with only 2.2%/3.1% of them were former smokers/drinkers and 0.5%/0.3% of them were current smokers/drinkers at baseline. Therefore, we were not able to adjust these two variables. Potential modifying effects of ER status, TNM stage, menopausal status, age at diagnosis (<65/≥65 years old), physical activity (Yes/No) and comorbidity (Yes/No) were tested using a multiplicative scale. Multiplicative interaction term was examined through likelihood ratio test, which compared the model including only main effects (reduced model) and the model including both main effects and interactive terms (full model). Further stratified analyses were conducted by ER status and menopausal status. All analyses and graphs were performed using SAS (version 9.4; SAS Institute, Cary, NC). RCS was completed by SAS macro %RCS^44^. Tests of statistical significance were two-sided, and *P* < 0.05 was considered as significant.

### Data Availability

The datasets generated and analyzed during the current study are available from the corresponding author on reasonable request.
